# Tetracycline Resistance Genes in the Traditional Swedish Sour Herring *surströmming* as Revealed Using qPCR

**DOI:** 10.3390/genes14010056

**Published:** 2022-12-24

**Authors:** Vesna Milanović, Antonietta Maoloni, Luca Belleggia, Federica Cardinali, Cristiana Garofalo, Cristiana Cesaro, Lucia Aquilanti, Andrea Osimani

**Affiliations:** Dipartimento di Scienze Agrarie, Alimentari ed Ambientali, Università Politecnica delle Marche, via Brecce Bianche, 60131 Ancona, Italy

**Keywords:** fermented fish, *tet*(M), *tet*(S), antibiotic resistance genes, traditional food

## Abstract

Antibiotic resistance (AR) represents a global concern for human health. To the best of the authors’ knowledge, no study addressing AR in *surströmming*, a traditional Swedish fermented herring, has been performed to date. The aim of the present research was to study the prevalence of *tet*(O), *tet*(S), *tet*(W), *tet*(K), and *tet*(M) genes encoding for resistance to tetracycline using quantitative PCR (qPCR) applied to ready-to-eat *surströmming* samples collected from three producers located in Sweden. The *tet*(M) gene was found in all the analyzed samples, and it was also the most abundant among the tested *tet* genes; moreover, *tet*(O) was the least frequently detected gene. As a general trend, all the analyzed samples showed a high occurrence of the target genes, with slight variations among the producers. A principal component analysis did not reveal any separation among the samples or producers. All the collected data allowed for a drawing of a first picture of the occurrence of tetracycline resistance genes in ready-to-eat *surströmming* samples. Since no differences among the samples manufactured by the different producers were observed, it is likely that the detected genes were homogeneously spread among the microbial species shared by the herrings used as raw materials. Moreover, it can be hypothesized that the presence of the detected genes was also the result of a selective pressure of the natural marine environment on the herrings’ gut microbiota and, hence, on the pro-technological microorganisms responsible for the fermentation of *surströmming*. However, the contribution of the manufacturers to the contamination of the processed herrings cannot be excluded.

## 1. Introduction

Fermented foods represent high-quality sources of nutrients. Moreover, the fermentation process allows for an increase in the safety and quality of food to be obtained, thus leading to end products characterized by peculiar textures, flavors, and biological functionalities [1]. Fermented foods can be obtained through the addition of so-called microbial starter cultures or through the metabolic activity of microorganisms naturally occurring in the raw materials [2]. Indeed, the microbiota of fermented foods are usually characterized by the presence of microorganisms that use the nutrients present in the raw materials to produce organic acids, which are mainly represented by lactic and acetic acids, together with other organic acids, such as formic, pyruvic, orotic, citric, uric, propionic, and butyric acids [3]. The production of these compounds allows for a reduction in pH to be obtained, with a consequent effect on spoilage and pathogenic microorganisms, which are killed or strongly inhibited. Hence, fermented foods are generally considered to be safe. Notwithstanding, among the safety features of a fermented food that must be considered, the occurrence of antibiotic-resistant bacteria is an ever-present issue. The antibiotic resistance (AR) of bacteria can be intrinsic or acquired; this latter phenomenon is usually triggered by bacterial mutation, or it is produced through the exchange of plasmids encoding for AR genes potentially transferable to commensal or pathogenic bacteria [4]. Moreover, although with a low magnitude, the occurrence of cell-free DNA can lead to gene transfer between a donor and recipient bacteria, even without temporal or physical contact [5]. As recently reported by the Organisation for Economic Co-operation and Development (OECD), the European Centre for Disease Prevention and Control (ECDC), the European Food Safety Authority (EFSA), and the European Medicine Agency (EMA), despite decreases in antibiotic consumption in both humans and food-producing animals, AR in bacteria from humans in the European Union and the European Economic Area has increased since 2011, as a kind of a silent pandemic [6]. Most worrying is the increase in resistance to critically important antibiotics used to treat common healthcare-associated infections [6]. As reported by the EFSA and the ECDC in the European Union Summary Report on Antimicrobial Resistance in zoonotic and indicator bacteria from humans, animals, and food in 2018/2019, sulfamethoxazole and tetracyclines are still recognized as highly important antibiotics, with bacterial resistance to tetracyclines being documented among those commonly observed [7]. Hence, the monitoring of AR in food represents a valid strategy for risk assessment and prevention. Regarding fermented foods, the AR issue emerged a long time ago in fermented meat, cheese, fermented vegetables, and even fermented fish [2,8,9,10].

*Surströmming* is a traditional Swedish food produced via the fermentation of herrings (*Clupea harengus* var. *membras*) [11]. The preparation of *surströmming* is carried out by pre-salting the herring in a saturated salt solution; then, the heads and entrails of the fish are removed, whereas the gonads (roe) and the pyloric ceca are retained. Herrings are left to ferment in a 17% salt brine in sealed barrels for up to 3 months at 15–18 °C [11,12]. Hence, the peculiar fermentation conditions applied during the production of *surströmming* select for the growth of well-adapted microbial species. To date, only a few studies have investigated the microbiota involved in the fermentation of *surströmming*. Indeed, to the best of the authors’ knowledge, two very recent studies conducted by Belleggia et al. [11] and Belleggia et al. [13] are the first to shed light on the microbiology of such a fermented fish product. In more detail, the study conducted by Belleggia et al. [11] revealed the presence of active microbial populations in ready-to-eat *surströmming*, mainly represented by mesophilic aerobes and mesophilic lactobacilli and lactococci, as well as halophilic lactobacilli and lactococci, coagulase-negative staphylococci, and halophilic aerobes and anaerobes. Interestingly, very low counts of Enterobacteriaceae and Pseudomonadaceae were observed by Belleggia et al. [11], whereas no sulfite-reducing anaerobes were detected. Among the core microbiota, *Halanaerobium praevalens*, *Alkalibacterium gilvum*, *Carnobacterium* spp., *Tetragenococcus halophilus*, *Clostridiisalibacter* spp., and Porphyromonadaceae were detected [11]. Although detected as a minority taxon, *Arcobacter* spp. was also found [11], suggesting potential safety concerns for consumers.

To the best of the authors’ knowledge, no studies addressing AR in *surströmming* have been performed, yet. Hence, the aim of the present research was to study the prevalence of *tet*(O), *tet*(S), *tet*(W), *tet*(K), and *tet*(M) genes encoding for resistance to tetracyclines by using the quantitative PCR (qPCR) method applied to the ready-to-eat *surströmming* samples previously studied by Belleggia et al. [11].

## 2. Materials and Methods

### 2.1. Sampling and DNA Extraction

Fifteen whole unpasteurized and canned fermented *surströmming* samples were bought from 3 different Swedish producers (5 samples per producer). The samples were labeled as follows: S1–S5 for samples bought from producer A, S6–S10 for samples bought from producer B, and S11–S15 for samples bought from producer C. A detailed description of the samples was previously provided by Belleggia et al. [11]. Aliquots (25 g) of each sample was tenfold diluted in sterile peptone water and homogenized at 260 rpm for 5 min using a Stomacher 400 Circulator (VWR International PBI, Milan, Italy). Double DNA extractions were performed for each sample from the cell pellets obtained after the centrifugation of the 1.5 mL aliquots of 10^−1^ homogenates using an E.Z.N.A. soil DNA kit (Omega Bio-tek, Norcross, GA, USA). The quantity and the purity of the extracted DNA were examined using a Nanodrop ND 1000 (Thermo Fisher Scientific, Wilmington, DE, USA) and PCR targeting the bacterial 16S rRNA gene using the universal prokaryotic primer pair 27F-1495R, as previously described by Belleggia et al. [11].

### 2.2. qPCR Quantification of Tetracycline Resistance Genes

Standard curves were created using decimal serial dilutions of the DNA extracted from 5 bacterial reference strains, each carrying one of the *tet* genes under study. In detail, *Enterococcus faecalis* TO15a [*tet*(M)], *Enterococcus italicus* 1102 [*tet*(S)], and *Lactobacillus casei/paracasei* ILC2279 [*tet*(W)] were obtained from the Culture Collection of the Department of Agricultural, Food and Environmental Sciences (D3A), whereas *Staphylococcus aureus* COL [*tet*(K)] and *Streptococcus pyogenes* 7008 [*tet*(O)] were obtained from the Culture Collection of the Department of Life and Environmental Sciences (DiSVA), both from the Polytechnic University of Marche (Ancona, Italy). Moreover, the reference strains carrying the known copy number of each tested gene were used as positive controls in qPCR reactions. The DNA extraction, quantification, PCR/qPCR amplification, and gene copy number calculations were performed as previously detailed by Milanović et al. [14]. The qPCR reactions were performed using a CFX Connect Real-Time System machine (Biorad, Hercules, CA, USA), which automatically calculated the qPCR amplification efficiencies (E) and correlation coefficients (R^2^) from the slopes of the standard curves. The standard curves were created in a range from <1 to 7 Log gene copies per reaction to estimate the qPCR detection limit for each tested *tet* gene. The absolute quantification of the *tet* genes was performed by running the DNAs extracted from the *surströmming* samples along with the tenfold serial dilutions of the qPCR standards. The qPCR mixtures were composed of (i) 5 μL of Type-it 2X HRM PCR Master Mix (Qiagen, Hilden, Germany); (ii) 0.45 μL of the primers (900 nM) (biomers.net GmbH, Ulm, Germany) used for the amplification of *tet*(K), *tet*(M), *tet*(O), and *tet*(S) genes and 0.2 μL of the primers (400 nM) (biomers.net GmbH) used for the amplification of the *tet*(W) gene; (iii) 4 μL of the DNA extracted from the samples; and (iv) molecular biology-grade water (SERVA Electrophoresis GmbH, Heidelberg, Germany) to reach a final volume of 10 μL. The qPCR primers were designed by Florez et al. [15], except for those that were used for the amplification of the *tet*(W) gene, which were designed by Wang et al. [16]. The thermal cycling conditions and melt curve analyses were the same as those previously described by Milanović et al. [17]. The slopes of the standard curves were used for the automatic calculation of each gene copy number per reaction. The results were expressed as the Log of the average gene copy number per gram of sample (4 replicates each: 2 DNA extractions × 2 qPCR technical replicates each) ± standard deviation.

### 2.3. Statistical Analyses

A one-way analysis of variance (ANOVA) among the samples was performed using Tukey–Kramer’s Honest Significant Difference (HSD) test (*p* < 0.05). A principal component analysis (PCA) using a correlation matrix was accomplished, utilizing the Log gene copy number per gram of sample for each AR gene as a variable, to evaluate the differences between the producers. A statistical analysis was carried out using the software JMP Version 11.0.0 (SAS Institute Inc., Cary, NC, USA).

## 3. Results

The effective extraction of the bacterial DNA from all the samples was confirmed by obtaining amplicons of the expected length (1468 bp). The automatic analysis of the qPCR standard curves resulted in R^2^ values >0.99 for all the tested *tet* genes, and E values of 93.3 and 100.6% were identified for *tet*(M) and *tet*(S) genes, respectively. The detection limit for *tet*(O), *tet*(S), and *tet*(W) was <1 Log gene copies per reaction, whereas the detection limit for *tet*(K) and *tet*(M) was 1 Log gene copy per reaction.

**Figure 1:**
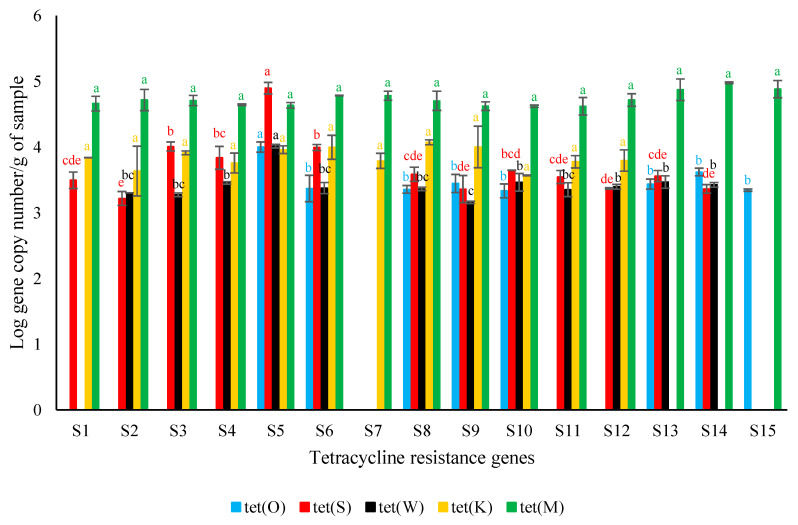
Average tetracycline resistance genes’ Log copy numbers per g of *surströmming* sample (S1–S5 (producer A), S6–S10 (producer B), and S11–S15 (producer C)) ± standard deviation. For each target gene, different letters indicate significant differences among samples (*p* < 0.05).

The qPCR results are reported in Figure 1.

*Tet*(M) was the only gene found in all the analyzed samples, being also the most abundant among the tested *tet* genes. *Tet*(O) was the least frequently detected gene and was found in 8 of the 15 samples herein analyzed. In more detail, the quantity of *tet*(M) was not significantly different among the samples, showing Log gene copy number per g of sample ranging from 4.62 ± 0.02 (S10, producer B) to 4.98 ± 0.01 (S14, producer C).

*Tet*(K) was detected in all the samples from producer A and producer B and in only two samples from producer C, without significant differences among the samples. The Log gene copy number per g of sample of *tet*(K) ranged from 3.63 ± 0.38 (S2) to 3.96 ± 0.06 (S5), both samples from producer A, and between 3.57 ± 0.01 (S10) and 4.07 ± 0.04 (S8), both samples from producer B. Samples S11 and S12 from producer C showed Log gene copy number per g of sample of 3.78 ± 0.09 and 3.79 ± 0.16, respectively.

*Tet*(W) was detected in 4 out of the 5 samples from each producer. The significantly lowest Log gene copy number per g of sample (3.16 ± 0.02) was exhibited by sample S9 (producer B), whereas the highest Log gene copy number per g of sample (4.01 ± 0.03) was showed by the S5 sample (producer A).

*Tet*(S) was found in all the analyzed samples, except for S7 (producer B) and S15 (producer C). The lowest Log gene copy number per g of sample (3.22 ± 0.11) was measured in S2, whereas the highest Log gene copy number per g of sample (4.89 ± 0.09) was detected in S5, both samples from producer A.

Finally, *tet*(O) was detected in only one sample (S5) from producer A; in all the samples from producer B, except for S7; and in samples S13, S14, and S15 from producer C. The significantly highest Log copy number per g of sample of this gene (4.00 ± 0.08) was found in S5, which was generally characterized by the highest amount of all the assayed genes.

The results of the PCA are depicted in Supplementary Materials. In more detail, the first component accounted for 44.7% of the variance, whereas the second component accounted for 30.3% of the variance. The samples from the three producers were not completely separated. In more detail, most of the samples from producer A (S2, S3, S4, and S5) and producer B (S6, S8, S9, and S10) were characterized by a positive relationship with positive loadings in Principal Component 1 (PC1) [*tet*(W), *tet*(S), and *tet*(K)], whereas two samples from producer C (S11 and S12) were characterized by positive loadings in PC1 [*tet*(W), *tet*(S), and *tet*(K)], and the remaining three samples (S13, S14, and S15) were characterized by positive loadings in Principal Component 2 [*tet*(M), *tet*(O), *tet*(W), and *tet*(S)].

## 4. Discussion

Tetracyclines are well-known antibiotics used for their wide-ranging spectrum of activity against Gram-positive and Gram-negative bacteria [18]. Bacterial resistance to tetracyclines is generally produced through the acquisition of mobile genetic elements carrying specific *tet* genes, mutations within the ribosomal binding site, and/or chromosomal mutations [18]. Among the specific mechanisms of resistance to tetracyclines, ribosomal protection, enzymatic inactivation, and efflux pumps have been reported [18]. Of note, the horizontal exchange of resistance genes can be due to mobile genetic elements, such as plasmids [19,20].

In the present study, all the analyzed samples showed a high occurrence of the tested *tet* genes. A high occurrence of transferable *tet* genes has previously been observed in enterococci isolated from fermented fish sampled from retail markets in Northeast India [21] and in lactic acid bacteria isolated from *pla-chom*, a fermented fish manufactured in Thailand [9]. Hence, it is estimated that marine aquaculture fish and the marine environment can be potential hot spots for the spread of AR genes, including those conferring resistance to tetracyclines [22]. Notwithstanding, the information on AR genes occurring in fermented fish products is still scarce.

In the present study, *tet*(M) was detected at the highest Log gene copy numbers per gram in all the samples, irrespective of the producer. The *tet*(M) gene encodes for a ribosomal protection protein implicated in tetracycline resistance [23]. This is the most frequently detected AR gene in both Gram-positive and Gram-negative bacteria inhabiting clinical, terrestrial, and marine environments [24]. *Tet*(M) was also the most detected AR gene in water-borne enterococci isolated from seawater and sediment from a Mediterranean aquaculture site where no antibiotics were used [25].

As for *tet*(S), this gene encodes for production of an elongation factor, which protects the ribosome from tetracycline binding [24]. Interestingly, Kim et al. [24] reported that aquaculture sites could be an important reservoir of *tet* genes, as both *tet*(M) and *tet*(S) were discovered by the same authors in fish intestinal and seawater bacteria isolated from coastal aquaculture sites.

*Tet*(K) encodes for the tetracycline efflux protein and is usually detected in Gram-positive bacteria [26,27], whereas *tet* (O) encodes for ribosomal protection [28]. It is difficult to make a further comparison of the data because, to the best of the authors’ knowledge, there is scarce information in the scientific literature on the occurrence of these genes in the aquatic environment.

Finally, *tet*(W) encodes for ribosomal protection, and its presence in the marine environment has already been reported by Nøhr-Meldgaard et al. [29] and Suzuki et al. [30].

Regarding the possible sources of the AR genes herein studied, it can be hypothesized that the detected genes could have been harbored by the bacteria (whether pro-technological or pathogenic) naturally contaminating the raw material (e.g., fish flesh) used to produce the *surströmming*; moreover, the occurrence of free DNA from the extracellular environment cannot be excluded [17].

Of note, the microbiota of the *surströmming* samples herein analyzed were previously subjected to a metataxonomic analysis by Belleggia et al. [11]; hence, the results regarding the *tet* genes obtained in the present study can be compared with the microbial taxa detected by Belleggia et al. [11], thus allowing correlations to be hypothesized. In such an investigation, a core microbiota dominated by *H. praevalens*, *A. gilvum*, *T. halophilus*, *Carnobacterium* spp., *Clostridiisalibacter* spp., and *Porphyromonadaceae* was found [11]. For most of these taxa, resistance to tetracyclines has previously been documented.

*T. halophilus*, formerly classified as *Pediococcus halophilus* and later reclassified as *Tetragenococcus*, is a Gram-positive bacterium that produces lactic acid from glucose [31]. To date, *T. halophilus* has mainly been isolated from salted and fermented foods, including fermented fish and fish sauces; despite its ability to improve the quality and sensory traits of these foods, *T. halophilus* has not yet acquired Qualified Presumption of Safety (QPS) qualification. Regarding the occurrence of *tet* genes in this bacterial species, Le Neindre et al. [23] reported that 26 out of 49 *T. halophilus* isolates collected from *doenjang*, a Korean high-salt-fermented soybean paste, showed a minimum inhibitory concentration (MIC) to tetracyclines higher than the breakpoint (4 mg L^−1^) provided by the European Committee on Antimicrobial Susceptibility test for the phylogenetically closely related species *Enterococcus faecium* and *E. faecalis*.

Even for *Carnobacterium*, a study examining the profiles of tetracycline-resistant commensal bacteria from representative ready-to-eat foods sampled from salad bars at grocery stores and restaurants in Columbus, Ohio, indicated this genus as a carrier of *tet* genes [32]. In a more recent study describing the bacterial communities and the AR genes of farmed rainbow trout fillet, samples displaying a higher *Carnobacterium* abundance were characterized by detectable *tet* genes [33]. Analogously, in a further recent study [34], an isolate ascribed to *Carnobacterium maltaromaticum* associated with the skin-associated mucus of salmonids sampled from the high Arctic region of Nunavut, Canada, showed multi-resistance to two or more antibiotics, including tetracyclines.

Analogously, a recent study investigating the distribution of AR genes in commensal chicken gut bacteria detected *tet* genes in the genomes of the family Porphyromonadaceae [35].

A few minority taxa were also detected by Belleggia et al. [11] in the surströmming samples herein analyzed, being *Psychrobacter celer*, *Marinilactibacillus psychrotolerans*, *Streptococcus infantis*, *Salinivibrio costicola*, Ruminococcaceae, and *Arcobacter* [11].

As for *Psychrobacter*, resistance to tetracycline has been observed in species of this genus isolated from a 15,000–35,000-year-old permafrost subsoil sediment collected from the Siberian Sea [36]. Interestingly, *Psychrobacter* spp. isolated from the internal organs of wild and commercial snow crabs (*Chionoecetes opilio* and *Chionoecetes japonicus*) showed resistance to tetracyclines [37], thus confirming fishery products as a source of bacteria resistant to this class of antibiotics.

Regarding *Marinilactibacillus*, Zhang et al. [38] have recently reported a correlation between the presence of *tet* genes and the occurrence of this bacterial genus in alfalfa silage, thus suggesting that *Marinilactibacillus*, together with other detected taxa in the same substrate, might carry these genes [38].

*S. infantis* was also observed among the minority taxa in the *surströmming* samples studied by Belleggia et al. [11] and herein analyzed. Interestingly, Ciric et al. [39] isolated a minocycline-resistant strain of *S. infantis*; the same authors also demonstrated that the *tet* gene conferring resistance to the isolated *S. infantis* strain was able to excise from the plasmid, with significant implications for the diffusion of such a *tet* gene [39].

*Salinivibrio costicola* can colonize hypersaline ecosystems, such as *surströmming*. Interestingly, Huang et al. [40], who described *S. costicola* subsp. *vallismortis* subsp. nov., also observed a natural resistance to many antibiotics, including tetracycline.

As recently reported by Juricova et al. [35], members of Ruminococcaceae isolated from chicken gut were shown to be carriers of three different *tet* genes, including *tet*(W).

*Arcobacter* was also found by Belleggia et al. [11] as a minority taxon in all the *surströmming* samples herein analyzed. The presence of this microorganism can represent a potential safety issue for consumers since *Arcobacter* can be the causative agent of different gastrointestinal diseases [41]. Interestingly, an analysis of genetic determinants encoding for tetracycline resistance in *Arcobacter* isolates obtained from different aquatic sources has revealed the presence of *tet*(O) and *tet*(W), thus representing a potential threat to human health [42].

Regarding the occurrence of AR genes in the form of free DNA from the extracellular environment, it is known that bacteria are able to insert free nucleic acid into their genomes during transformation [43]. Several bacterial species, being naturally transformable or competent, have already been demonstrated to possess the genes involved in DNA uptake [43]. Interestingly, Calderón-Franco et al. [44] have recently demonstrated that, although AR gene uptake via natural transformation is not likely to be the main transfer mechanism, even a small amount of water free-floating extracellular DNA can be taken up by naturally competent bacteria in complex ecosystems via transformation processes.

As reported by Belleggia et al. [11], the production of *surströmming* is usually realized with herrings caught in the Northern regions of the Baltic Sea (Gulf of Bothnia). Of note, as reported by the Baltic Marine Environment Protection Commission [45], tetracyclines have been applied in aquaculture, mariculture, and agriculture with input into the Baltic Sea, thus leading to downstream impacts on resistance genes in samples collected from different sites of the Baltic Sea. Interestingly, Gross et al. [46] have recently isolated tetracycline-resistant *Escherichia coli* from marine mammals and various fish species of the North and Baltic Seas, thus evidencing the role of the resident fish as “sentinels” for the AR phenomenon. Moreover, Muziasari et al. [47], who analyzed the composition of the antibiotic resistome from the intestinal contents of fish from Baltic Sea farms, observed that the analyzed resistomes included *tet* genes, thus contributing to the enrichment of AR genes in sediments below fish farms in the Northern Baltic Sea.

## 5. Conclusions

All the collected data allowed for a drawing of a first picture of the occurrence of *tet* genes in ready-to-eat *surströmming*. Since no significant differences among the samples manufactured by the different producers were observed, it is likely that the detected genes were homogeneously spread among the microbial species shared by the herrings used as raw materials. However, the contribution of the manufacturers to the contamination of the processed herrings cannot be excluded.

Based on the available literature on the spread of *tet* genes in microorganisms harbored by fish species inhabiting the Northern Baltic Sea [46,47], it is likely that the presence of the detected genes is also the result of a selective pressure of the natural marine environment on the herrings’ gut microbiota and, hence, also on the pro-technological microorganisms responsible for the fermentation of *surströmming*.

In the present study, the relatively low number of samples collected from each producer could represent a limitation to the generalization of the results. However, the results on the occurrence of *tet* genes herein obtained undoubtedly improve the knowledge on the risk of AR occurring in this ready-to-eat fermented food, whose magnitude has never been assessed before, thus supporting the need for continuous monitoring and further investigation, including the isolation of microorganisms on selective growth media containing antibiotics.

## Data Availability

Not applicable.

## Supplementary Materials

The following supporting information can be downloaded at: https://www.mdpi.com/article/10.3390/genes14010056/s1, Results of principal component analysis (PCA) performed on the *surströmming* samples according to tetracycline resistance genes (*tet*(O), *tet*(S), *tet*(W), *tet*(K), and *tet*(M)). Samples labeled in red (from S1 to S5) are from producer A, samples labeled in green (from S6 to S10) are from producer B, and samples labeled in blue (from S11 to S15) are from producer C.
