# Supplementary material for: Tetracycline Resistance Genes in the Traditional Swedish Sour Herring surströmming as Revealed Using qPCR
Source: Genes (Basel). 2022 Dec 24;14(1):56. doi: 10.3390/genes14010056 (PMC9858948; doi:10.3390/genes14010056)

Supplementary material. Results of principal component analysis (PCA) performed onto the *surströmming* samples according to tetracycline resistance genes [*tet*(O), *tet*(S), *tet*(W), *tet*(K), and *tet*(M)]. Samples labelled in red (from S1 to S5) are from producer A, samples labelled in green (from S6 to S10) are from producer B, and samples labelled in blue (from S11 to S15) are from producer C.

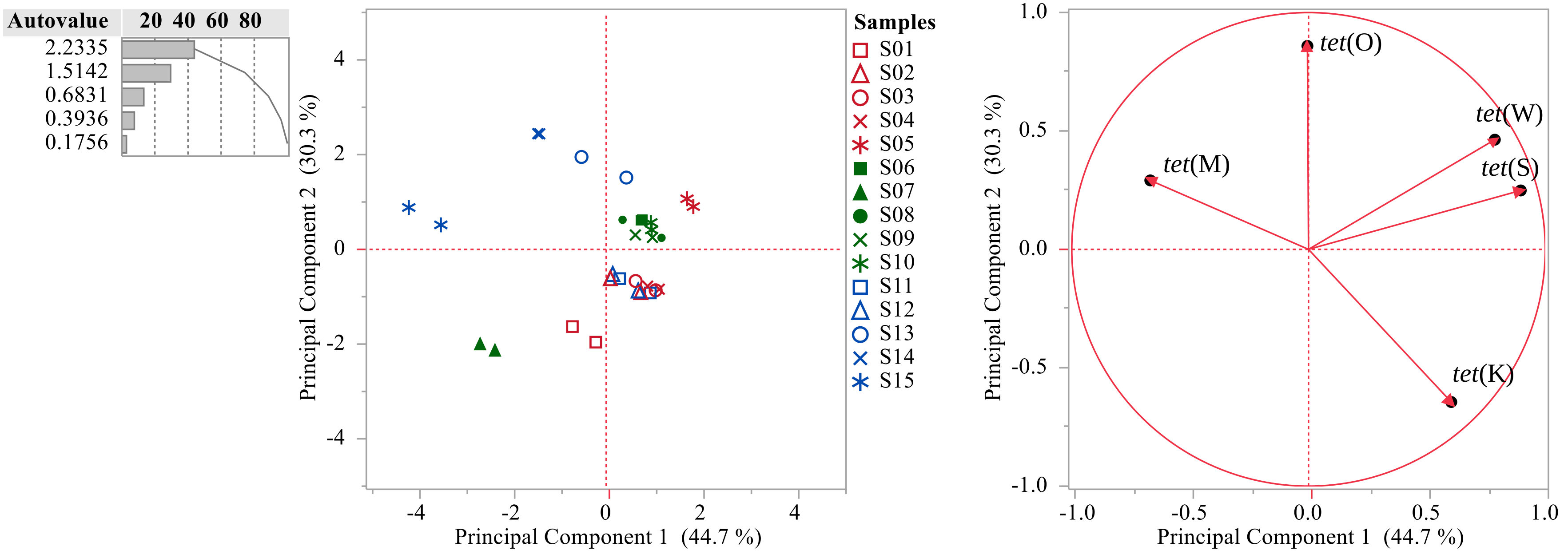

Supplement: Supplementary file 1 [file genes-14-00056-s001.zip › genes-2117336-supplementary.pdf]
